# Formulation of a generalised switching CFAR with application to X-band maritime surveillance radar

**DOI:** 10.1186/s40064-015-1347-2

**Published:** 2015-10-05

**Authors:** Graham V. Weinberg

**Affiliations:** National Security and Intelligence Division, Defence Science and Technology Group (DSTG), Edinburgh, SA 5111 Australia

**Keywords:** Radar detection, CFAR, Switching CFAR, X-band clutter, Pareto type distributions

## Abstract

A generalisation of a switching based detector is examined, allowing the construction of such detectors for target detection in any clutter model of interest. Such detectors are important in radar signal processing because they are robust solutions to the management of interference. Although formulated in general terms, the theory is applied to the design of a switching constant false alarm rate detector for X-band maritime surveillance radar. It is shown that such a detector manages the problem of interference better than standard detection processes.

## Background

**Fig. 1:**
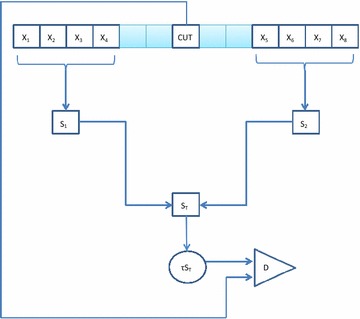
Structure of a CFAR Process. The clutter statistics $X_1, X_2, \ldots , X_8$ are combined to produce $S_T,$ which is then normalised by $\tau$ and compared to the CUT in the decision stage (D). Two guard cells are used on either side of the CUT

Constant false alarm rate (CFAR) detectors are of considerable importance in radar signal processing, and as such, have been the focus of much research over the years (Goldstein 1973; Nitzberg 1979; Gandhi and Kassam 1988; Blake 1988; Shor and Levanon 1991; Anastassopoulos and Lampropoulos 1995; Nagle and Saniie 1995; Cao 2008; Meng 2009; Erfanian and Vakili 2009; Tablet and Soltani 2009; Pourmottaghi et al. 2012; Qin et al. 2013; Zhang et al. 2013; Weinberg 2013, 2014; Zhang 2015). In this paper, CFAR is understood to be in the context as outlined in Gandhi and Kassam (1988), and as illustrated in Fig. 1. It is assumed that a series of independent and identically distributed clutter amplitude or intensity measurements are available, known as the clutter range profile, from which a measure of the clutter level is taken. This is then normalised and compared to a cell under test (CUT). In the example illustrated in Fig. 1 the clutter statistics are denoted $X_1, X_2, \ldots , X_8,$ and the two processes denoted $S_1$ and $S_2$ are applied to the two subsets of the clutter range profile to allow for separate measurements of the clutter to be combined to produce a single measurement $S_{T}.$ A number of guard cells are used to separate the CUT from the clutter range profile as illustrated. The normalised measure of clutter is $\tau S_{T}.$ If the CUT exceeds this normalised measurement a target is declared present (Minkler and Minkler 1990). The normalisation is used so that the false alarm probability remains constant. Such detectors are desirable because variations in clutter power can have serious consequences on detection performance, such as increased false alarms resulting in false detections, and missed detection of real targets due to thresholds set too high in practice (Minkler and Minkler 1990).

Although the theory of CFAR detectors is well developed for the Exponentially distributed clutter case, there has been a requirement to re-examine the theory of CFAR as radar resolution has improved with corresponding advances in radar hardware design and processing throughput, resulting in improvements in radar resolution. Finer radar resolution is at the expense of spikier clutter returns, and an inevitable deviation from the validity of the Exponentially distributed clutter assumption (Shor and Levanon 1991; Anastassopoulos and Lampropoulos 1995). Consequently there has been much work on designing CFAR processes for more appropriate clutter models (Pourmottaghi et al. 2012; Weinberg 2013).

In the context of airborne X-band maritime surveillance radar, this is certainly the case, and recent work has established the Pareto clutter model assumption as valid (Farshchian and Posner 2010; Weinberg 2011). Thus there has been much research devoted to the design of CFAR detectors under a Pareto clutter model assumption (Weinberg 2013, 2014b).

Switching detectors were first examined in Cao (2008), which developed a switching CFAR based upon the cell-averaging (CA)-CFAR for target detection in Exponentially distributed clutter. It is shown in Cao (2008) that the performance of the CA-CFAR can be improved in the presence of interference through a switching process. This approach has thus generated much interest and stimulated ongoing research into the technique’s refinement and application to other clutter environments (Meng 2009; Erfanian and Vakili 2009; Tablet and Soltani 2009; Zhang et al. 2013).

The purpose of this paper is to show how the application of a switching CFAR to Pareto clutter introduced in Weinberg (2014b) can be used to formulate a general switching detector for application to any clutter model of interest. The key feature of a switching detector is that it pre-processes the clutter range profile to censor anomalous measurements of clutter. The approach in Weinberg (2014b) shows that the statistical structure of this censored clutter range profile, for the Pareto case, is equivalent to that as formulated in Cao (2008), for the Exponentially distributed clutter case. It will be shown that this can be generalised further, so that the censored set can be specified so that the properties of it for the Pareto case can be applied to an arbitrary clutter model.

Based upon this, a generalised switching detector is formulated, and a general expression for its probability of false alarm is derived, which can then be used to set threshold multipliers in practice. To illustrate this, a new switching detector is examined, based upon a Lomax distribution, which has become of much interest to researchers at Defence Science and Technology Group (DSTG) as a potential alternative to the standard Pareto model.

The paper is organised as follows “CFAR and transformed detectors” outlines CFAR from a mathematical perspective, and also overviews an appproach to the construction of CFARs based upon transformations. “Switching detector formulation” formulates the general switching CFAR, while “Example of switched detector” proposes a new switching CFAR based upon the Lomax clutter model assumption. “Ingara data and distributional fit” discusses the data set under consideration, including clutter model fitting. Finally “Application to X-band radar” provides some examples of detector’s performance, under a Lomax clutter model assumption, relative to non-switched detectors.

## CFAR and transformed detectors

To begin, standard noncoherent CFAR processing is discussed in a mathematical framework. Useful references on this approach are Gandhi and Kassam (1988) and Minkler and Minkler (1990). Assume the independent and identically distributed clutter statistics are $X_1, X_2, \ldots , X_N$ and the statistic of the CUT is *X*,  which is also assumed to be independent of each member of the clutter range profile. A general detection scheme can be specified by

1 $$\begin{aligned} X \mathop{\gtrless}\limits_{H_{0}}^{H_{1}} g(X_1, X_2, \ldots , X_N; \tau ), \end{aligned}$$

where the known function *g* computes a measure of the average level of clutter, and $\tau$ is a threshold factor, which is used to regulate the false alarm probability. The notation used in (1) means that the null hypothesis is rejected if and only if $X > g(X_1, X_2, \ldots , X_N; \tau ).$ The probability of false alarm (Pfa) of (1) is given by

2 $$\begin{aligned} \mathrm{\mathbf Pfa}= \mathbb{P}(X > g(X_1, X_2, \ldots , X_N; \tau ) | H_0), \end{aligned}$$

and $\tau$ is obtained, for a given Pfa, by inversion of (2). In the case where $\tau$ does not vary with the clutter power, the detection process (1) is said to have the CFAR property (Minkler and Minkler 1990), and (1) is referred to as a CFAR detector. In the case of Exponentially distributed clutter, there are many such processes that are CFAR. Two key ones are the CA-CFAR

3 $$\begin{aligned} X \mathop{\gtrless}\limits_{H_{0}}^{H_{1}} \sigma \sum _{j=1}^N X_j \end{aligned}$$

and the order statistic (OS)-CFAR

4 $$\begin{aligned} X \mathop{\gtrless}\limits_{H_{0}}^{H_{1}} \nu X_{(k)}, \end{aligned}$$

for some $1 \le k \le N,$ where it can be shown through (2) that $\sigma = \mathrm{\mathbf Pfa}^{-1/N}-1$ and $\nu$ is set via numerical inversion of

5 $$\begin{aligned} \mathrm{\mathbf Pfa}= \frac{ N!}{(N-k)!} \frac{\Gamma (N-k+\nu + 1)}{\Gamma (N+\nu + 1)}, \end{aligned}$$

where $\Gamma (\cdot )$ is the Gamma function (Gandhi and Kassam 1988).

These detectors can be transformed to operate in a clutter environment of interest using the transformation approach of Weinberg (2014), which generalised the Pareto case in Weinberg (2013). The elegant feature of this method is the detector is modified while the threshold multiplier ($\sigma$ or $\nu$) is still set via the original Pfa expression. This method will be used to modify (3) and (4) to operate in Lomax distributed clutter in “Example of switched detector”; here we outline the general transformation approach for completeness.

Suppose we are interested in a clutter environment modelled by a random variable *Y*,  with distribution function $F_Y(t):=\mathrm{{I\ P}}(Y\le t).$ In order to adapt the detection process (1) to operate in clutter modelled by random variables $Y_1, Y_2, \ldots , Y_N$ with distribution function $F_Y,$ one introduces a transfer function

6 $$\begin{aligned} H(t) := F_{Y}^{-1} \left( 1-e^{-t}\right) , \end{aligned}$$

which can be shown to be monotonic and hence possesses a unique inverse. Then the decision rule

7 $$\begin{aligned} \widehat{Y} \mathop{\gtrless}\limits_{H_{0}}^{H_{1}} H\left( \tau g\left( H^{-1}(Y_1), H^{-1}(Y_2), \ldots , H^{-1}(Y_N)\right) \right) \end{aligned}$$

is a transformed version of (1), where $\widehat{Y}$ is the CUT and $\tau$ is set via (2). Thus the transformation approach allows the generation of detectors from those designed to operate in Exponentially distributed clutter. As illustrated in the Pareto clutter case in Weinberg (2013), the transformation approach may result in loss of the CFAR property with respect to particular clutter parameters.

## Switching detector formulation

Although this paper is focused on switching detectors, it is worth commenting on the fact that these are not the only solution to managing irregularities in clutter for CFAR processes. In particular, Smith and Varshney (1997) introduced a variability index (VI)-CFAR algorithm. This detector, designed to operate in independent Exponentially distributed clutter returns, has the CFAR property and is shown to manage interfering targets well. Additionally it is shown that it regulates the Pfa reasonably well. This detector partitions the clutter range profile into two sets, and runs a test to see whether there is variability in the two. It then tests to see whether the means of the two differ. Based upon these tests, and appropriate variation of the CA-CFAR is selected. An investigation revealed that when applied to the Pareto case, this detector becomes dependent on knowledge of the Pareto shape parameter, and hence is not a CFAR detector for the case of Pareto distributed clutter returns. Furthermore, even with the application of an maximum likelihood estimator for the Pareto shape parameter, the algorithm becomes computationally expensive to run in comparison to a switching detector.

The idea behind a switching detector is to determine whether any of the statistics in the clutter range profile can be classified as being irregular. Such irregularities in measurements could be due to clutter power level increases or spurious interfering targets. These irregularities, once identified, are censored from the detection process. Suppose $S_0$ is a subset of the clutter range profile, consisting of clutter returns deemed not irregular. It will be shown how to select $S_0$ so that the statistical properties of it in the Pareto case can be applied to arbitrary clutter models.

Let $g_{|S_0}$ be the restriction of the clutter measurement function *g* to the set $S_0.$ To illustrate this, suppose *g* is a sum, with clutter range profile $\{Y_1, Y_2, \ldots , Y_N\}$ and that $S_0 = \{Y_1, Y_3, Y_5\}.$ Then $g_{|S_0}(Y_1, Y_2, \ldots , Y_N) = Y_1 + Y_3 + Y_5.$ If instead, *g* was an order statistic, then $g_{|S_0}(Y_1, Y_2, \ldots , Y_N)$ would be the corresponding order statistic on the set $\{Y_1, Y_3, Y_5\}.$

The general form of a switching-based detector, based upon (1), can be formulated as follows. A target is declared present in the CUT (denoted $\widetilde{Y}$) if one of the two conditions are met:

8 $$\begin{aligned} \widetilde{Y}& > g_{|S_0}(Y_1, Y_2, \ldots , Y_N; \kappa ) \text{ when } n_0 > N_T \nonumber \\ \nonumber \\ \widetilde{Y} &> g(Y_1, Y_2, \ldots , Y_N; \kappa ) \,\,\, \,\, \text{ when } n_0 \le N_T, \end{aligned}$$

where $\kappa >0$ is a threshold factor, $n_0$ is the size of the set $S_0$ and $N_T \in \{1,2,\ldots , N\}$ is an integer threshold constant. As in Cao (2008) appropriate selection for $N_T$ can be based upon the need to manage up to $N_I$ expected interfering targets, so that $N_T = N - N_I -1$ as justified in Cao (2008) is utilised.

It is clear that it is vital to determine a useful way in which to specify the set $S_0.$ Once this is done, it is then possible to determine $\kappa$ through an expression for the Pfa of (8).

In the case of Exponentially distributed returns, the set $S_0$ is defined by $S_0 = \{Z_j: Z_j < a Z\}$ where $Z_j$ are the clutter statistics, *Z* is the CUT and $a>0$ is a fixed constant Cao (2008). Selection of parameter *a* is described comprehensively in Cao (2008) and Weinberg (2014b). This set can be used as a basis for all switching detectors with a simple application of the inverse of (6). The key to this is to reformulate the set $S_0$ so that it is in terms of clutter statistics for the desired clutter model. To see this, it is shown in Weinberg (2014) that if *Z* is an Exponentially distributed random variable with unity mean then *H*, defined in (6), has the property that $H(Z) \mathop {=}\limits ^{d}Y$ and $Z \mathop {=}\limits ^{d}H^{-1}(Y),$ where *Y* is the desired clutter model. Hence an application of these to the Exponentially distributed clutter range profile, and under the assumption of $H_0,$

9 $$\begin{aligned} S_0 &= \{Z_j : Z_{j} < a Z\} \equiv \left\{ Y_j : H^{-1}(Y_j) < a H^{-1}(Y)\right\} \nonumber \\ & = \left\{ Y_j: Y_j < H \left( a H^{-1}(Y)\right) \right\} , \end{aligned}$$

where $Z_j = H^{-1}(Y_j)$ for all *j*. Thus, (9) suggests an appropriate way in which clutter statistics may be sorted for a switching detector, based upon the clutter model of interest. Observe that if the set $S_0$ is determined through (9), then any properties of $S_0$ under $H_0$ that have been established can be applied directly to the clutter environment of interest. In particular, it is shown in Weinberg (2014b) that the distribution of $n_0$ under $H_0$ is given by

10 $$\begin{aligned} \mathbb{{P}}(n_0 = k | H_0) = {N \atopwithdelims ()k} a^{-1} B\left( N-k+ a^{-1}, k+1\right) , \end{aligned}$$

where $k \in \{0, 1, \ldots , N\}$ and $B(\cdot , \cdot )$ is the Beta function.

Finally, it is necessary to specify the Pfa of (8), to produce the threshold factor $\kappa.$ In the detection scheme (1), since it is assumed that the clutter statistics are independent and identically distributed, suppose that $\mathrm{\mathbf Pfa}(n)$ denotes the false alarm probability when there are *n* clutter statistics. Then, since clutter is sorted via (9), it follows by conditional probability that

11 $$\begin{aligned} \mathrm{\mathbf Pfa}&= \sum _{k=0}^{N_{T}} \mathbb{P}\left( \widetilde{Y} > g_{|S_0}(Y_1, Y_2, \ldots , Y_N; \kappa ) | H_0\right) \nonumber \\&\quad \times \mathbb{P}(n_0 = k | H_0) \nonumber \\&\quad + \sum _{k=N_T + 1}^N \mathbb{P}\left( \widetilde{Y} > g(Y_1, Y_2, \ldots , Y_N; \kappa )|H_0\right) \nonumber \\&\quad \times \mathbb{P}(n_0 = k | H_0) \nonumber \\&= \mathrm{\mathbf Pfa}(N) \mathbb{P}(n_0 \le N_T | H_0) \nonumber \\&\quad + \sum _{k=N_T + 1}^N \mathrm{\mathbf Pfa}(k) \mathbb{P}(n_0 = k | H_0). \end{aligned}$$

Consequently, one can determine $\kappa$ via numerical inversion of (11). Hence (8), together with (9), (10) and (11), provide a generalised switching detector. Clearly if the detector on which it is based is CFAR with respect to a certain clutter parameter, then the switching detector will inherit this property. The switching detector parameter *a*,  used in the definition of $S_0,$ can also be selected based upon the guidelines established in Weinberg (2014b).

## Example of switched detector

Many of the clutter intensity models of interest in X-band maritime surveillance radar can be represented in the form

12 $$\begin{aligned} F_Y(t) = 1 - e^{-\mu h(t)} \end{aligned}$$

where $\mu > 0$ is the clutter shape parameter, and *h*(*t*) has the properties that $h(0) = 0$ and it is increasing, so that (12) is a well-defined distribution function. To illustrate this, the choice of $h(t) = t$ produces the Exponential distribution, $h(t) = t^n$ (with $n>0$) results in the Weibull family and $h(t) = \log (t/\beta )$ yields a Pareto model. By applying (12) to (6), it can be shown that $H(t) = h^{-1}(t/\mu )$ and consequently $H^{-1}(t) = \mu h(t).$ Thus by applying these to (9), it follows for the model (12) that

13 $$\begin{aligned} S_0 = \left\{ Y_j : Y_j < h^{-1}\left( a h(\widetilde{Y})\right) \right\} , \end{aligned}$$

providing a general form for the set $S_0$ in terms of function *h*, which is applicable for any clutter model based upon (12).

To illustrate (9), consider the case where *Y* has a Pareto distribution with shape parameter $\alpha$ and scale parameter $\beta.$ Then its distribution function is

14 $$\begin{aligned} F_{Y}(t) = 1 - \left( \frac{\beta }{t}\right) ^\alpha , \end{aligned}$$

provided $t \ge \beta.$ Hence $H(t) = \alpha \log (t/\beta )$ and so $H^{-1}(t) = \beta e^{ t/\alpha }.$ Hence, applying these to (9), it can be shown that $S_0 = \{Y_j: Y_j < \beta ^{1-a} Y^a\},$ which is exactly the same set as in Weinberg (2014b).

Although the Pareto distribution has been shown to fit real X-band clutter returns very well, there are issues arising from the Pareto scale parameter present in the detectors examined in Weinberg (2013). It has been found that the presence of this parameter results in poorer detection performance. Examples of this are apparent in the numerical analysis in Weinberg (2014b), where the Pareto scale parameter is estimated adaptively. Hence an alternative to the two-parameter Pareto model has also been investigated at DSTG, where the clutter is assumed to follow the one-parameter Pareto or Lomax model with distribution function

15 $$\begin{aligned} F_{Y}(t) = 1 - \left( \frac{1}{1+t}\right) ^\mu \end{aligned}$$

where $t \ge 0.$ This distribution has $h(t) = \log (1+t)$ in the formulation (12), and hence $H(t) = e^{t/\mu }-1$ based upon the above analysis. Consequently, one can apply this with the transformed detector (7) to produce new decision rules to operate in clutter modelled by (15). Based upon the analysis in “CFAR and transformed detectors”, the detector (3) transforms to

16 $$\begin{aligned} \widetilde{Y} \mathop{\gtrless}\limits_{H_{0}}^{H_{1}} \prod _{j=1}^N \left( Y_j + 1\right) ^\sigma - 1, \end{aligned}$$

with $\sigma$ set as for (3). Similarly, the detector (4) becomes

17 $$\begin{aligned} \widetilde{Y} \mathop{\gtrless}\limits_{H_{0}}^{H_{1}} \left( Y_{(k)}+1\right) ^\nu -1, \end{aligned}$$

with $\nu$ set via (5).

Both detectors (16) and (17) are CFAR with respect to the clutter shape parameter. Consequently, a switching detector based upon these will also preserve the CFAR property. Here we focus on a switched version of (16). It can be shown easily via (13) that a switching detector based upon this clutter model will necessitate $S_0 = \{Y_j : \log (1 + Y_j) < a \log (1+\widetilde{Y})\}.$ The form of the switched detector is to reject $H_0$ if

18 $$\begin{aligned} \widetilde{Y}> & {} \prod _{Y_j \in S_0}\left( Y_j + 1\right) ^\kappa - 1 \text{ when } n_0 > N_T \nonumber \\ \widetilde{Y}> & {} \prod _{j=1}^N \left( Y_j + 1\right) ^\kappa - 1 \,\,\, \,\, \text{ when } n_0 \le N_T, \end{aligned}$$

with $\kappa$ determined via (11) with $\mathrm{\mathbf Pfa}(k) = (1+\kappa )^{-k}.$

Before examining the performance of the detector, a brief overview of the DSTG Ingara data is considered, together with an analysis of the Pareto and Lomax fits to this data.

## Ingara data and distributional fit

DSTG’s Ingara radar has provided researchers with X-band high resolution medium to high grazing angle polarimetric clutter returns that have assisted in the analysis of radar detection schemes (Stacy et al. 1996). This radar has been deployed in a series of trials, and subsequent analysis of the resultant clutter and detection performance has been documented extensively (Stacy et al. 2005; Crisp et al. 2007; Rosenberg 2010).

The clutter set used for the following analysis consists of a series of pure clutter returns obtained through a trial conducted by DSTG in 2004, and is designated run 34,683. The Ingara radar operated at X-Band, with a centre frequency of 10.1 GHz, and uncompressed pulse width of 20 μs, and range resolution of 0.75 m. The trial was run in the Southern Ocean, roughly 100 km south of Port Lincoln in South Australia. Further details of the Ingara radar can be found in Stacy et al. (1996), while a summary of the trial, and analysis of the resultant radar clutter, can be found in Stacy et al. (2005), Rosenberg (2010). The data was obtained through a number of runs, where in each such run the full 360° of azimuth angles were scanned. Thus the radar operated in a circular spotlight mode.

Analysis of Ingara data, and in particular the Pareto model fit, can be found in Weinberg (2011). Run 34,683 consists of 840,704 clutter returns, from the data set in the approximate up wind direction. This data set is thus the most spiky, and hence is the worst case scenario from a detection perspective. Only horizontal transmit and receive, and vertical transmit and receive, polarisations are considered. This is due to the fact that the vertically polarised case corresponds to approximate Rayleigh clutter amplitude statistics, while the horizontal polarised case consists of spikier clutter returns that are non-Rayleigh distributed (Weinberg 2011). Cross polarisation clutter tends to be distributed between these two extremes, and so the two polarisations considered represent the two extreme cases of clutter.

Statistical modes were fitted to these data sets via maximum likelihood estimation algorithms. In the fitting of the Pareto distribution to the horizontally polarised data set yielded estimates of $\alpha = 4.7241$ and $\beta = 0.0446,$ while in the vertically polarised case, these were $\alpha = 11.3930$ and $\beta = 0.3440.$ The Lomax distribution resulted in a shape parameter of $\mu =84.8173$ for horizontal polarisation, and $\mu = 31.2739$ in vertical polarisation.

**Fig. 2:**
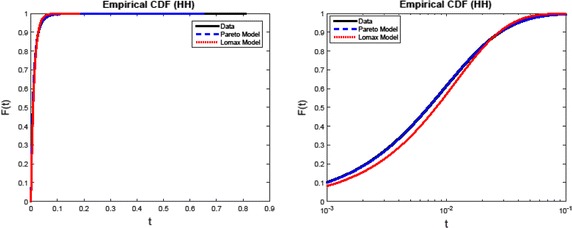
Empirical distribution fit to Ingara data set under consideration, with horizontal polarisation. The* plot* shows the data distribution function, the corresponding Pareto and Lomax fits. The* right subplot* is a magnification of the* left subplot*, and shows the slight discrepancy in the Lomax fit

**Fig. 3:**
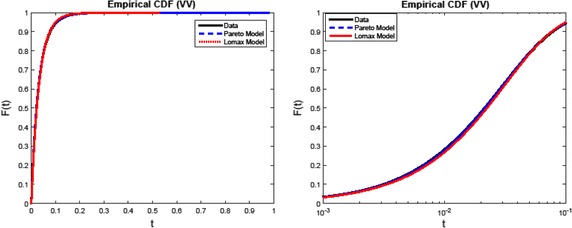
Plot similar to that in Fig. 2, except in the vertically polarised case. Here the discrepancy in fit is smaller than in the horizontally polarised case

To examine the validity of the model fits, Figs. 2 and 3 plot empirical distribution functions of the Ingara data sets, together with that for the Pareto and Lomax models. In the case of horizontal polarisation, Fig. 2 shows that the Pareto fit is very good, while that of the Lomax model has a slight error. This is attributable to a one-parameter model. Figure 3 shows that in the vertically polarised case, the Lomax fit improves considerably.

The next section examines the performance of (18) relative to (16) and (17).

## Application to X-band radar

It is of interest to investigate whether the switching detector (18) improves on the performance of (16) when subjected to interference. Additionally, it is of interest to assess the performance of (18) relative to (17), since OS-based CFARs are robust to interference (Gandhi and Kassam 1988). This is done by simulating clutter from the model (15), where $\mu$ is matched to estimates obtained from real radar clutter.

For the detector analysis, $N=32$ with the Pfa set to 10^−4^. The SW-CFAR uses $a=1.5$ and $N_{T} = 29,$ while the OS-CFAR uses $k=30.$ The latter two choices are selected so that the respective detector can manage up to two interfering targets. Examining performance in both these polarisations enables guidelines on detection strategies to be formulated. Detection performance is estimated in all cases using $10^6$ Monte Carlo samples for each signal to clutter ratio (SCR).

**Fig. 4:**
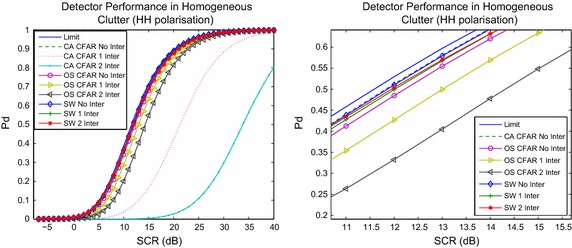
Performance of the three detectors in homogeneous horizontally polarised clutter, and with up to two interfering targets. The* right subplot *is an enlargement of a component of the* left subplot*, to examine performance in closer detail. Detector (16) is referred to as CA-CFAR in this figure, while (17) is called OS-CFAR to reflect the fact that they are CFAR detectors. The switching detector (18) is denoted SW in the figure. If subjected to interference, the respective detector is marked appropriately as outlined in the legend

**Fig. 5:**
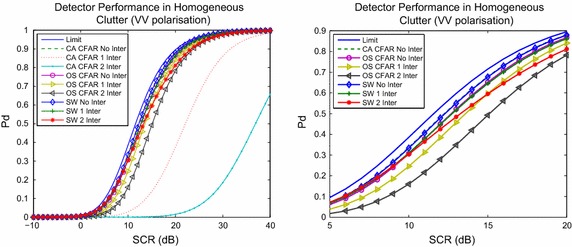
Analysis of detector performance in vertically polarised clutter. Detectors are marked in the legend as for the previous figure. Markings of detectors as for the previous two figures

Figure 4 shows detection performance in the horizontally polarised clutter case. The plot shows detection performance with a Swerling 1 target model applied to the CUT. Additionally, the three detectors are subjected to up to two Swerling 1 interfering targets, whose SCR are 20 and 30 dB respectively. The figure also plots a limit, based upon (3). The detector (16) is referred to as CA-CFAR, while (17) is called OS-CFAR, to reflect the decision rules on which they are based. The detector (18) is referred to as SW as shown. The right subplot is a magnification of the left in a subset of SCR values. The figure shows that the switching based detector has the best performance in terms of managing the strong interference. Specifically, the switching detector is better than that based upon an order statistic by around 0.3 dB (for no interference), by 0.7 dB for the case of 1 interfering target and by approximately 2 dB for the case of two interfering targets. The CA-CFAR, on which the switching detector is based, has the worst performance by contrast. Figure 5 illustrates the same scenario, except under vertical polarisation, showing a similar performance to that in Fig. 4.

Next, it is important to estimate the resultant Pfa from each of the new detection processes, since they are designed in the case of uniform clutter to maintain the desired Pfa. To do this, clutter was simulated as for the detection performance curves, and the resultant Pfa was measured via Monte Carlo estimation with $10^6$ runs. Additionally, the resultant Pfa was measured when the clutter range profile was subjected to an independent interfering Swerling 1 target model, with SCR of 5 dB. Table 1 plots the resultant Pfa. Recalling that the design Pfa has been set to 10^−4^, we see that in the case of no interference, the switching detector tends to improve on the detector (16) in both polarisations. In the horizontally polarised case, the switching detector has the largest deviation from the design Pfa. However, in the vertically polarised case, the switching detector is best at maintaining the desired Pfa.

**Table 1:** Pfa estimates in uniform clutter, including when detectors are subjected to interference, through the insertion of a 5 dB Swerling 1 target into the clutter range profile

	HH		VV	
	No inter	Inter	No inter	Inter
CA-CFAR	0.94 × 10^−4^	5.5 × 10^−5^	1.07 × 10^−4^	5.5 × 10^−5^
OS-CFAR	1.06 × 10^−4^	4.2 × 10^−5^	1.15 × 10^−4^	4 × 10^−5^
SW	0.91 × 10^−4^	5.1 × 10^−5^	1.01 × 10^−4^	5.3 × 10^−5^

The design Pfa is 10^−4^

In the case of interference, the detector (17) performs the best in maintaining the desired Pfa. The switching detector only slightly improves on the performance on the detector (16). It is interesting to note that in the presence of interference, the resultant Pfa is smaller than the design Pfa. From a practical perspective, this is better than having an increase in the false alarm rate.

**Fig. 6:**
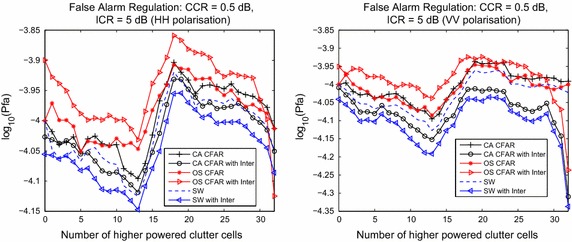
False alarm regulation for the three detectors, including the case where an interfering target is present in the clutter range profile. The* left subplot* is for horizontal polarisation, while the* right* corresponds to vertical polarisation

To explore further the performance of these detectors in managing the Pfa, their performance is assessed during clutter transitions or false alarm regulation. False alarm regulation studies the effect on the resultant Pfa as the number of clutter cells in the clutter range profile are slowly increased with higher powered clutter returns (Gandhi and Kassam 1988). Figure 6 plots the estimated Pfa as a function of the number of higher powered clutter cells, for the three detectors under consideration. The power level increase is known as the clutter to clutter ratio (CCR), and has been set to 0.5 dB. In addition to this, an interfering target has been inserted into the clutter range profile to examine the effect on the estimated Pfa. This is a Swerling 1 target model as before, and its SCR has been set to 5 dB. This is known as the interference to clutter ratio (ICR). Monte Carlo runs with $10^6$ samples have been used to produce the plots in Fig. 6. The left subplot is for horizontal polarisation, while the right corresponds to the vertically polarised case. The figure shows that the detectors tend to manage the false alarm regulation in a similar way. What is notable is that the SW-CFAR tends to reduce the number of false alarms in the presence of interference, while the OS-CFAR tends to allow more false alarms. When the mid-point of the clutter range profile is completely saturated with higher powered returns, the CUT is then considered to be affected by this, which is why there is a characteristic jump in the plots after 16 cells are affected.

The results shown in Figs. 4, 5 and 6 demonstrate that the switching detector has a greater capacity to manage interfering targets than the non-switched detector on which it is based. It also has slightly better performance than an OS based detector. These results are common to the two polarisations. In terms of false alarm regulation, the switching detector tends to reduce the number of false alarms, especially in the presence of interference, as Fig. 6 shows.

## Conclusions

A general formulation of a switching detector was presented, and it was shown how the set $S_0$ could be selected so that it became equivalent to that used for the case of Exponentially distributed clutter. This meant that setting the threshold multiplier for a switching detector could be done in a uniform way regardless of the underlying clutter model. Consequently, this generalised the Pareto case in Weinberg (2014b).

Due to issues with the Pareto clutter model, a Lomax distribution was examined, and a switched version of a transformed CA-CFAR was analysed. It was shown to provide a robust solution to the management of interference. Furthermore, its performance exceeded that of the non-switched equivalent, as well as that of an OS-CFAR. Further work will examine the application of such a detection scheme directly to real data.
